# Li–S Chemistry of Manganese Phosphides Nanoparticles With Optimized Phase

**DOI:** 10.1002/advs.202207470

**Published:** 2023-02-03

**Authors:** Qiao Deng, Xinji Dong, Pei Kang Shen, Jinliang Zhu

**Affiliations:** ^1^ School of Resources Environment and Materials Collaborative Innovation Center of Sustainable Energy Materials State Key Laboratory of Featured Metal Materials and Life‐cycle Safety for Composite Structures Guangxi University Nanning 530004 P. R. China

**Keywords:** heterostructure, lithium–sulfur batteries, manganese phosphide, phase evolution, porous carbon

## Abstract

The targeted synthesis of manganese phosphides with target phase remains a huge challenge because of their various stoichiometries and phase‐dependent physicochemical properties. In this study, phosphorus‐rich MnP, manganese‐rich Mn_2_P, and their heterostructure MnP–Mn_2_P nanoparticles evenly dispersed on porous carbon are accurately synthesized by a convenient one‐pot heat treatment of phosphate resin combined with Mn^2+^. Moreover, their electrochemical properties are systematically investigated as sulfur hosts in lithium–sulfur batteries. Density functional theory calculations demonstrate the superior adsorption, catalysis capabilities, and electrical conductivity of MnP–Mn_2_P/C, compared with MnP/C and Mn_2_P/C. The MnP–Mn_2_P/C@S exhibits an excellent capacity of 763.3 mAh g^−1^ at 5 C with a capacity decay rate of only 0.013% after 2000 cycles. A phase evolution product (MnS) of MnP–Mn_2_P/C@S is detected during the catalysis of MnP–Mn_2_P/C with polysulfides redox through in situ X‐ray diffraction and Raman spectroscopy. At a sulfur loading of up to 8 mg cm^−2^, the MnP–Mn_2_P/C@S achieves an area capacity of 6.4 mAh cm^−2^ at 0.2 C. A pouch cell with the MnP–Mn_2_P/C@S cathode exhibits an initial energy density of 360 Wh kg^−1^.

## Graphical abstract

Phosphorus‐rich MnP, manganese‐rich Mn_2_P, and their heterostructure MnP–Mn_2_P nanoparticles are accurately synthesized and their electrochemical properties and enhancement mechanisms are systematically investigated as sulfur hosts in Li—S batteries.

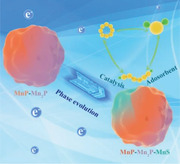

## 1 Introduction

Lithium–sulfur (Li–S) batteries are promising rechargeable energy systems with sulfur as the positive electrode, which are expected to replace lithium‐ion batteries owing to their supernal theoretical specific capacity (1675 mAh g^−1^), excellent energy density (2600 Wh kg^−1^), low cost, and low environmental impact.^[^ ^1^ ^]^ However, the large‐scale practical application of Li–S batteries is limited by the following shortcomings:^[^ ^2^ ^]^ i) sluggish reaction kinetics, resulting from electronic/ionic insulating ability of sulfur; ii) electrode structure degradation for huge volumetric variations (≈80%) between sulfur and Li_2_S; and iii) low utilization of sulfur and capacity fade caused by the so‐called “shuttle effect”, that is, intermediate lithium polysulfides (LiPSs) dissolve in the electrolyte and migrate to the anode. To overcome these issues, extensive effort has focused on enhancing Li–S batteries, such as physical modification,^[^ ^3^ ^]^ chemisorption,^[^ ^4^ ^]^ and catalysis.^[^ ^5^ ^]^ Carbon materials, such as carbon nanotubes,^[^ ^6^ ^]^ graphene,^[^ ^7^ ^]^ and porous carbon,^[^ ^8^ ^]^ are the most common sulfur hosts owing to their high electronic conductivity, which improves the conductivity of sulfur and physical adsorption of LiPSs.^[^ ^9^ ^]^ Nevertheless, the weak interaction of LiPSs with non‐polar carbon materials decreases the effectivity of carbon materials in inhibiting the shuttle effect of soluble polysulfides.^[^ ^10^ ^]^

Polar metal compounds, including metal oxides,^[^ ^11^ ^]^ nitrides,^[^ ^12^ ^]^ sulfides,^[^ ^13^ ^]^ and phosphides,^[^ ^14^ ^]^ have been extensively studied as polar hosts owing to their abundant polar active sites and inhibition of polysulfide shuttling through strong chemical bonding interactions, thereby enhancing the stability of electrochemical cycling.^[^ ^15^ ^]^ This results in the efficient chemisorption of polysulfides, higher electrical conductivity, and good electrocatalysis for the redox reaction of LiPSs.^[^ ^16^ ^]^ In 2017, Wang et al.^[^ ^17^ ^]^ first demonstrated the fast kinetics for LiPSs redox conversion reaction and stable cycling performance using transition metal phosphides (molybdenum phosphide nanoparticles). Qian et al.^[^ ^18^ ^]^ systematically investigated the kinetic behaviors of cobalt‐based compounds for LiPSs conversion, whereby cobalt phosphide possessed the lowest overpotential, compared with Co_3_O_4_, CoS_2_, and Co_4_N. Thus, among the aforementioned suggested compounds, transition metal phosphides are effective.

Manganese, a transition metal element with lower cost, abundant yield, and hypotoxicity, has been garnering considerable interest in Li–S batteries.^[^ ^19^ ^]^ Manganese‐based compounds, especially MnO_2_, are among the earliest and most famous polar compounds used as sulfur hosts.^[^ ^20^ ^]^ Wang et al. reported the ability of mesoporous MnO_2_/S composites to deliver a stable capacity of 1356.1 mAh g^−1^ at 0.5 mg cm^−2^ after 100 cycles.^[^ ^21^ ^]^ In addition, manganese single atom^[^ ^22^ ^]^ and manganese sulfide^[^ ^23^ ^]^ have strong chemical adsorption of LiPSs with high electrocatalytic activity for LiPSs redox reaction. Moreover, Yang et al. exhibited that Mn_2_P nanoparticles interact with the electrolyte in Li–S battery to facilitate rapid electron transport, thereby improving their electrochemical performance.^[^ ^24^ ^]^ Density functional theory (DFT) calculation indicates that MnP*_x_* dramatically catalyzes polysulfide conversion.^[^ ^25^ ^]^ Therefore, these studies inspired us to believe that manganese phosphides and their heterogeneous structures combining two distinct manganese phosphides could serve as excellent host candidates in Li–S batteries. Herein, to obtain manganese‐based phosphides with optimized phase, we synthesized a series of various stoichiometric Mn*_x_* P_y_ (MnP, MnP–Mn_2_P, and Mn_2_P) on carbon networks by adjusting the potassium hydroxide content during the heat treatment of phosphate resin–manganese species. Moreover, their electrochemical performances and phase evolution between manganese‐based phosphides and polysulfides in charging/discharging processes were systematically investigated for Li–S batteries.

## 2 Results and Discussion

**Figure 1:**
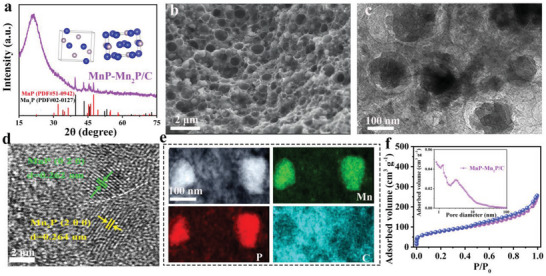
a) XRD, b) SEM image, and c,d) TEM and HRTEM images for MnP–Mn_2_P/C. d,e) STEM–HAADF and EDX element distribution mapping and f) N_2_ adsorption/desorption isotherm of MnP–Mn_2_P/C.

MnP/C, Mn_2_P/C, and MnP–Mn_2_P/C were obtained by controlling the manganese species concentrations with various stoichiometries via regulating KOH content during pyrolysis process. The MnP, MnP–Mn_2_P, and Mn_2_P contents in the prepared samples were 42.4 wt%, 48.4 wt%, and 54.9 wt%, respectively. The content of MnP in MnP–Mn_2_P/C was 19.6 wt%. The X‐ray diffraction (XRD) pattern of MnP–Mn_2_P/C (**Figure** **1**a; Figure S1, Supporting Information) shows a combination of MnP (JCPDS NO.51‐0924) and Mn_2_P (JCPDS No.02‐0127) peaks, indicating the presence of MnP and Mn_2_P. By adjusting the potassium hydroxide content, MnP/C and Mn_2_P/C were obtained. Scanning electron microscopy (SEM) and transmission electron microscopy (TEM) were used to explore the microstructure of the materials. MnP/C, Mn_2_P/C, and MnP–Mn_2_P/C exhibited 3D porous structures with filled macropores of several hundred nanometers (Figure 1b; Figure S2, Supporting Information). The SEM results of MnP–Mn_2_P/C revealed the presence of nanoparticles embedded in the porous structures. Moreover, a TEM image (Figure 1c) further exhibited their “honeycomb” porous characteristics, which provide sufficient space for sulfur and restrict polysulfide diffusion.^[^ ^26^ ^]^ The high‐resolution TEM image (Figure 1d) exhibits the lattice spacing of 0.262 and 0.264 nm, for the MnP (0 2 0) and Mn_2_P (2 0 0) planes, respectively. The MnP–Mn_2_P heterointerface, which promoted the anchoring of LiPSs and efficient electron transport in Li–S batteries as MoN–VN interface,^[^ ^27^ ^]^ was clearly observed. Scanning transmission electron microscopy–high–angle annular dark‐field image (STEM–HAADF) and energy‐dispersive X‐ray spectrometry (EDX) (Figure 1e) revealed the homogeneous element distributions of C, P, and Mn within MnP–Mn_2_P/C. The N_2_ adsorption/desorption isotherms of MnP–Mn_2_P/C, MnP/C, and Mn_2_P/C exhibit the existence of micropores and mesopores, as shown in Figure 1f; Figure S3, Supporting Information. The specific surface area of MnP–Mn_2_P/C was 255.85 m^2^ g^−1^, which was slightly higher than that of MnP/C (217.77 m^2^ g^−1^) and Mn_2_P/C (192.3 m^2^ g^−1^). The resulting large specific surface area and homogeneous pore size distribution could provide sufficient and efficient space for sulfur. In the Raman spectra (Figure S4, Supporting Information), the D and G bands of carbon coincided with the two characteristic peaks at 1340 and 1590 cm^−1^, respectively. The ratio of the D and G bands (*I*_D_/*I*_G_) indicated the graphitization and intrinsic disorder of the carbon matrix structures. In particular, the *I*_D_/*I*_G_ value for MnP–Mn_2_P/C (1.2) was slightly higher than that of MnP/C (1.1) and Mn_2_P/C (1.15), indicating its higher graphitization and improved electronic conductivity.^[^ ^28^ ^]^ In the X‐ray photoelectron spectroscopy (XPS) spectra, the position of P 2p plot (Figure S5a, Supporting Information) at 132.75 and 133.5 eV corresponded to the P–Mn and P–C peaks, respectively.^[^ ^29^ ^]^ The Mn 2p spectrum in MnP–Mn_2_P/C was convolved into three peaks at 640.95, 642.8, and 645.2 eV, which were ascribed to Mn 2p_3/2_ (Figure S5b, Supporting Information).^[^ ^30^ ^]^

MnP–Mn_2_P/C@S was prepared using a melt‐diffusion process. Typical XRD peaks associated with the Fddd orthorhombic of sulfur (JCPDS No.08–0247) were observed, as shown in Figure S6a, Supporting Information, indicating the successful loading of sulfur into MnP–Mn_2_P/C@S. The thermogravimetric analysis (TGA) obtained the sulfur content of 80.6% for MnP–Mn_2_P/C@S (Figures S6b and S7, Supporting Information). From the N_2_ adsorption/desorption isotherms and pore size distribution (Figures S6c and S8, Supporting Information), the specific surface area of MnP–Mn_2_P/C@S (18.95 m^2^ g^−1^) was significantly lower than that of the MnP–Mn_2_P/C, indicating that the sulfur particles occupied almost all the empty spaces in the pore structure. XPS results of MnP–Mn_2_P/C@S are similar as before sulfur loading (Figure S9, Supporting Information). MnP–Mn_2_P/C Sulfur nanoparticles were more uniformly loaded on the MnP–Mn_2_P/C@S surface without the agglomeration of large sulfur nanoparticles, as shown in the SEM and TEM images (Figures S6d and S6e, Supporting Information, respectively). An S (0 4 0) crystalline plane with a crystalline spacing of 0.32 nm was clearly observed in the HRTEM (Figure S6f, Supporting Information), suggesting the successful loading of sulfur nanoparticles in MnP–Mn_2_P/C. The STEM and elemental mapping (Figure S6g–k, Supporting Information) of MnP–Mn_2_P/C@S display the homogeneous distribution of the four elements within the composite, namely C, P, Mn, and S.

**Figure 2:**
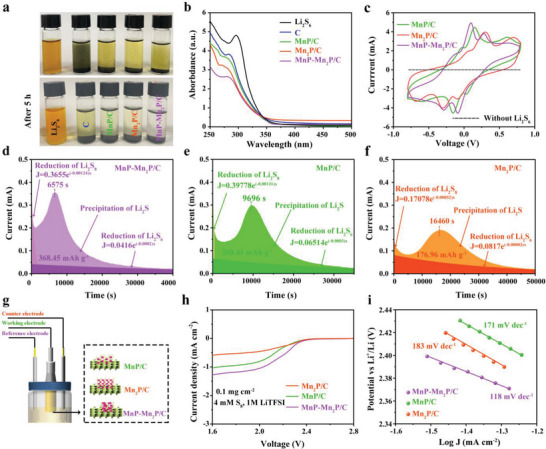
a) Visualized adsorption tests and b) optical absorbance of the Li_2_S_6_ solution after 5 h of MnP–Mn_2_P/C, Mn_2_P/C, MnP/C, and C. c) Typical CV curves of the symmetric cells at 5 mV s^−1^ of MnP–Mn_2_P/C, Mn_2_P/C, MnP/C, and MnP–Mn_2_P/C without Li_2_S_6_. Fitted current versus time curve for d) MnP–Mn_2_P/C, e) Mn_2_P/C, and f) MnP/C during potentiostatic discharge at 2.10 V. g) Schematic of the three‐electrode LSV system on different electrocatalysts of MnP–Mn_2_P/C, Mn_2_P/C, and MnP/C. h) LSV curves for the S_8_ reduction tested in a 4 mm S_8_ ether‐based electrolyte. i) Tafel plots calculated from the LSV curves in (h).

The ultraviolet–visible (UV–vis) spectroscopy and direct observation revealed the clear differences in the color of the solutions with different adsorbers, suggesting their different affinities to adsorb LiPSs after 5 h. The color of the solution changed after 5 h, with the MnP–Mn_2_P/C solution having the lightest color, as shown in **Figure** **2**a, indicating its rapid and strong adsorption affinity. MnP/C and Mn_2_P/C also exhibited a weaker adsorption affinity. However, color is only subjective to the human eye and is often disturbed by the color of the powder. From the UV–vis spectra (Figure 2b), Li_2_S_6_ has a strong absorption at 250–350 nm. Compared with MnP/C, Mn_2_P/C, and C, the absorbance for MnP–Mn_2_P/C in this region strongly decreased, indicating a stronger polysulfide adsorption capacity. To further explore the conversion ability of the samples, the CV profile of the symmetric cell was tested at a scan rate of 5 mV s^−1^ (Figure 2c). This process was achieved using MnP–Mn_2_P/C (Figure S10, Supporting Information), MnP/C (Figure S11, Supporting Information), and Mn_2_P/C (Figure S12, Supporting Information) as the working and counter electrodes, and 0.3 mol L^−1^ Li_2_S_6_ as the electrolyte. Compared to MnP/C and Mn_2_P/C, MnP–Mn_2_P/C exhibited the highest current intensities and narrowest peak separations, indicating its excellent electrocatalytic effect from a reaction kinetic point of view, thereby enhancing the sulfur conversion and reversibility. To analyze the advantages of the heterostructures for the conversion of polysulfides, nucleation experiments on Li_2_S were conducted using different catalytic materials. Figure 2d–f shows the nucleation experiments of Li_2_S to confirm the effect of MnP–Mn_2_P/C on the liquid–solid conversion rate. Compared to the MnP/C (260.41 mAh g^−1^, 9696 s) and Mn_2_P/C (176.96 mAh g^−1^, 16460 s), MnP–Mn_2_P/C exhibited the highest capacity (368.45 mAh g^−1^) and earliest responsivity (6575 s) with the sharpest peak shape, indicating the efficient facilitation of the conversion of polysulfides to insoluble Li_2_S and rapid kinetics as shown in Li_2_S nucleation morphologies (Figure S13, Supporting Information). To further confirm the excellent catalytic performance of MnP–Mn_2_P/C, a three‐electrode system was prepared by linear scanning voltammetry (LSV) using a counter, reference, and rotating disc electrodes (Figure 2g). Three‐electrode LSV revealed the highest current response of MnP–Mn_2_P/C, compared with MnP/C and Mn_2_P/C, indicating its excellent limiting current density during the polysulfide reduction process (Figure 2h). MnP–Mn_2_P/C exhibited the smallest Tafel slope (118 mV dec^−1^), compared with MnP/C (171 mV dec^−1^) and Mn_2_P/C (183 mV dec^−1^) (Figure 2i), indicating that MnP–Mn_2_P/C has an extremely fast kinetic rate of polysulfide conversion, alleviating polysulfide oxidation and promoting rapid electron transfer.^[^ ^31^ ^]^

**Figure 3:**
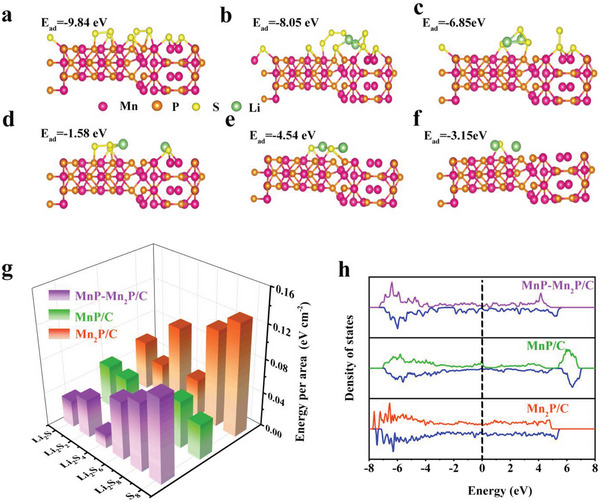
a–f) First‐principles calculation results, illustrating the chemical interactions among S_8_, Li_2_S_8_, Li_2_S_6_, Li_2_S_4_, Li_2_S_2,_ Li_2_S, and MnP–Mn_2_P/C. g) Calculated adsorption energies for Li_2_S*_x_* (*x* = 1, 2, 4, 6, and 8). h) DOS analysis of Mn_2_P/C, MnP/C, and MnP–Mn_2_P/C.

Density functional theory calculations were carried out using VASP to optimize the S_8_, Li_2_S_8_, Li_2_S_6_, Li_2_S_4_, Li_2_S_2_, and Li_2_S structures adsorbed on different surfaces to further demonstrate the adsorption ability of MnP–Mn_2_P/C. **Figure** **3**a–f shows the most stable adsorption configurations of the MnP (0 2 0)–Mn_2_P (2 0 0) heterostructures at S_8_, Li_2_S_8_, Li_2_S_6_, Li_2_S_4_, Li_2_S_2_, and Li_2_S, respectively. The rearrangement of MnP–Mn_2_P/C on the S_8_, Li_2_S_8_, Li_2_S_6_, Li_2_S_4_, Li_2_S_2_ and Li_2_S adsorption conformations with binding energies of 9.84, 8.05, 6.85, 1.58, 4.54, and 3.15 eV, respectively, justify its heterostructure (Figure 3g). Compared to MnP (Figure S14, Supporting Information) and Mn_2_P (Figure S15, Supporting Information) in Li_2_S_6_, MnP–Mn_2_P heterostructure had the highest binding energy, which agreed with the results of the adsorption experiments. Moreover, the MnP–Mn_2_P/C heterostructure exhibited a moderate adsorption energy per unit area, relative to MnP/C and Mn_2_P/C, indicating its excellent adsorption ability and effective facilitation of the polysulfide conversion.^[^ ^32^ ^]^ This is attributed to the empty orbitals in Mn, which can accept lone electron pairs from the Lewis bases to form coordination bonds, resulting in Lewis acid–base interactions that can interact to anchor the polysulfide.^[^ ^25^ ^]^ Thus, the MnP–Mn_2_P heterostructure can significantly inhibit the solubilization of the polysulfide. Figure 3h shows the electronic density of states (DOS) of the MnP/C, Mn_2_P/C, and MnP–Mn_2_P/C heterostructure obtained by theoretical calculations (Figure S16, Supporting Information). MnP–Mn_2_P/C had the highest DOS metal content at the Fermi energy level, compared to MnP/C and Mn_2_P/C, indicating its excellent electrical conductivity, effective improvement on the insulating properties of sulfur, and promoted rapid electron transfer.

**Figure 4:**
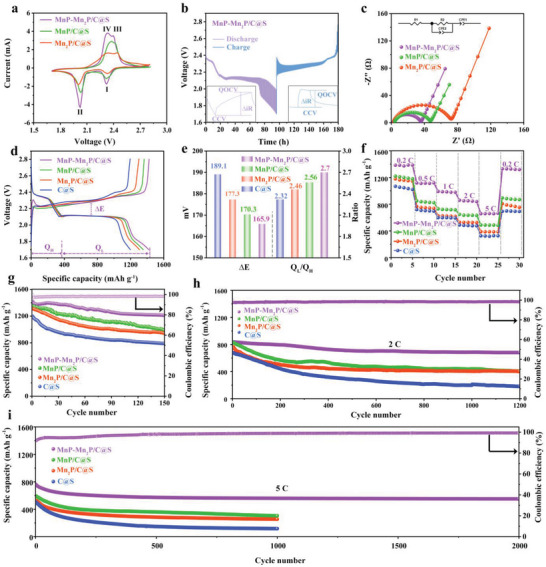
a) CV curves at the scan rate of 0.4 mV s^−1^. b) Galvanostatic intermittent titration (GITT) voltage profiles of MnP–Mn_2_P/C@S and c) Nyquist curves of MnP–Mn_2_P/C@S, Mn_2_P/C@S, and MnP/C@S. d) Charge/discharge profiles. e) Δ*E* and *Q*_L_/*Q*_H_ values obtained from the charge/discharge curves of the Li–S batteries. f) Rate performances at 0.2–5 C. g) Cycling performances at 0.1 C of MnP–Mn_2_P/C@S, Mn_2_P/C@S, MnP/C@S, and C@S. Long‐term cycling performance at h) 2 C and i) 5 C of MnP–Mn_2_P/C@S, Mn_2_P/C@S, MnP/C@S, and C@S.

To further evaluate the electrochemical performance of the electrodes, **Figure** **4**a shows the cyclic voltammetry curves (CV) of the Li–S cells based on MnP–Mn_2_P/C@S (Figures S17 and S18, Supporting Information), MnP/C@S (Figure S19, Supporting Information), and Mn_2_P/C@S (Figure S20, Supporting Information) cathodes within the voltage range of 1.7–2.8 V at a scan rate of 0.4 mV s^−1^. All curves displayed two reduction peaks at 2.31 V (peak I) and 2.07 V (peak II) in two steps. Peak I is associated with the reduction of sulfur to long‐chain LiPSs, whereas peak II is associated with the further reduction of LiPSs to insoluble Li_2_S_2_/Li_2_S.^[^ ^33^ ^]^ The two peaks of the anode at 2.4 V (peak III) and 2.34 V (peak IV) are associated with the multi‐step oxidation of Li_2_S_2_/Li_2_S to LiPSs to S_8_, respectively.^[^ ^34^ ^]^ The Li–S cell based on MnP–Mn_2_P/C@S cathode exhibited the highest current response, narrowest peak spacing (0.07 V), and highest/lowest onset potential for the reduction and oxidation peaks, respectively.

The lithium‐ion (Li^+^) diffusion rate, which is another factor that affects the LiPSs conversion kinetics, was investigated using the CV curves at variable rates of 0.1–0.5 mV s^−1^ for MnP–Mn_2_P/C@S, MnP/C@S, and Mn_2_P/C@S. Figure S21, Supporting Information, shows the reduction and oxidation peak currents for MnP–Mn_2_P/C@S, MnP/C@S, and Mn_2_P/C@S, which had a linear relationship with the square root of the scan rates. Thus, the lithium‐ion diffusion rate at different scan rates could be calculated using the classical Randles–Ševčík equation:^[^ ^35^ ^]^

(1) $$I_{P}=269\;000n^{1.5}AD_{{\mathrm{Li}}^{+}}^{0.5}C_{{\mathrm{Li}}^{+}}v^{0.5}$$

where *n* is the number of electrons transferred in the reaction, *A* is the electrode area, C_Li_^+^ is the lithium‐ion concentration in the electrolyte, D_Li_^+^ is the Li^+^ diffusion coefficient, and *v* is the scan rate. The slope of the peak current of MnP–Mn_2_P/C@S is steeper than that of MnP/C@S and Mn_2_P/C@S, indicating the highest Li^+^ diffusivity during the oxidation and reduction reactions of MnP–Mn_2_P/C@S.

To further determine the Li^+^ diffusion coefficient, galvanostatic intermittent titration (GITT) tests for MnP–Mn_2_P/C@S, MnP/C@S, and Mn_2_P/C@S are performed. As shown in Figure 4b, MnP–Mn_2_P/C@S exhibits the lowest internal resistance (∆*iR*) compared with MnP/C@S and Mn_2_P/C@S (Figure S22, Supporting Information). The highest Li^+^ diffusivity obtained for MnP–Mn_2_P/C@S reflects the more efficient catalytic activity toward Li–S reaction process and trapping of soluble LiPSs, thereby preventing them from dissolving into the electrolyte.^[^ ^14^ ^]^ Thus, MnP–Mn_2_P/C promotes the redox kinetics and increases the LiPSs conversion ability. Based on the equivalent circuit shown in Figure 4c, MnP–Mn_2_P/C@S has the lowest charge transfer and ohmic resistances. The lowest charge transfer of MnP–Mn_2_P/C@S is attributed to the significant decrease in its charge transfer resistance, facilitated Li^+^ transportation, and shortened Li^+^ diffusion pathway. Meanwhile, the lowest ohmic resistance of the MnP–Mn_2_P/C@S cathode of Li–S batteries can provide a fast Li^+^ diffusion pathway.

Figure 4d shows a comparison of the first charge/discharge cycle of the Li–S batteries with MnP–Mn_2_P/C@S, MnP/C@S, Mn_2_P/C@S, and C@S at a current density of 0.1 C in the voltage range of 1.7–2.8 V (vs Li^+^/Li). Two discharge plateaus appeared at approximately 2.30 and 2.07 V, corresponding to the two cathodic reduction peaks in the CV profiles, reflecting the S_8_ → LiPSs → Li_2_S_2_/Li_2_S reduction process. Two charge plateaus appeared at ≈2.34 and 2.4 V, corresponding to the two oxidation peaks in the CV profiles, reflecting the Li_2_S_2_/Li_2_S → LiPSs → S_8_ oxidation process.^[^ ^36^ ^]^ The initial discharge specific capacity of the Li–S cell using MnP–Mn_2_P/C@S cathode was 1419 mAh g^−1^ at 0.1 C, which is higher than that of MnP/C@S, Mn_2_P/C@S, and C@S (1353.5, 1309, and 1196.1 mAh g^−1^, respectively). MnP–Mn_2_P/C@S had the smallest polarization potential (∆*E* = 165.9 mV), compared with MnP/C@S, Mn_2_P/C@S, and C@S (*∆*E = 170.3, 177.3, and 189.1 mV, respectively), as shown in Figure 4e. This facilitated the redox reaction kinetics of MnP–Mn_2_P/C@S toward LiPSs conversion. In principle, *Q*_L_/*Q*_H_ is related to the electrocatalytic activity of the polysulfide conversion. A higher *Q*_L_/*Q*_H_ value increases the electrocatalytic performance.^[^ ^37^ ^]^ The Li–S batteries with MnP–Mn_2_P/C@S cathode had a *Q*_L_/*Q*_H_ value of 2.70, which is higher than those using MnP/C@S (2.56), Mn_2_P/C@S (2.46), and C@S (2.32). This demonstrates that MnP–Mn_2_P/C@S increases the catalytic activity and improves the redox kinetics of polysulfides.

The rate capabilities were further tested, as shown in Figure 4f. Compared with the specific capacities of MnP/C@S, Mn_2_P/C@S, and C@S (1210, 1160, and 1090 mAh g^−1^, respectively), MnP–Mn_2_P/C@S (Figure S23, Supporting Information) had the highest capacity of 1384.7 mAh g^−1^ at 0.2 C. The reversible capacity of MnP−Mn_2_P/C@S was 1118.3, 991, and 859.8 mAh g^−1^ at current densities of 0.5, 1, and 2 C, respectively. As the current density was increased from 0.5 C to 1 C to 2 C, the capacity loss rates of MnP/C@S, Mn_2_P/C@S, and C@S gradually improved, whereas MnP–Mn_2_P/C@S maintained a high and stable capacity. Furthermore, even at a higher current density of 5 C, MnP–Mn_2_P/C@S had a specific capacity of 657.9 mAh g^−1^. Notably, the cells using MnP–Mn_2_P/C@S cathode still exhibited excellent capacity retention even at high rates. When the current density was returned to 0.2 C, the discharge capacity of MnP–Mn_2_P/C@S was recovered at 1319 mAh g^−1^. The excellent rate performance demonstrated that MnP–Mn_2_P/C facilitates fast electron transfer channels and efficiently promotes polysulfide conversion.

The cycling stabilities of the Li–S batteries using various cathodes were carried out at 0.1 C with the sulfur loading of 1.8 mg cm^−2^. Figure 4g shows the cycling performance of MnP–Mn_2_P/C@S (Figure S24, Supporting Information), MnP/C@S (Figure S25, Supporting Information), Mn_2_P/C@S (Figure S26, Supporting Information), and C@S (Figure S27, Supporting Information) at 0.1 C. MnP–Mn_2_P/C@S had the highest initial capacity of 1419 mAh g^−1^, which was maintained at a capacity decay rate of 0.097% after 150 cycles, compared with MnP/C@S (1353.5 mAh g^−1^, 0.178% capacity decay rate), Mn_2_P/C@S (1309 mAh g^−1,^ 0.186% capacity decay rate), and C@S (1196.1 mAh g^−1^, 0.225% capacity decay rate). Thus, the excellent electrocatalytic properties of MnP–Mn_2_P/C@S can be further introduced to significantly improve the electrochemical performance of Li–S batteries. Moreover, the initial discharge capacity of MnP–Mn_2_P/C@S was 838 mAh g^−1^ at a current density of 2 C and the specific capacity could reach 681 mAh g^−1^ after 1200 cycles, as shown in Figure 4h. In addition, the initial discharge capacity of MnP–Mn_2_P/C was 763.3 mAh g^−1^ at a high current density of 5 C (Figure 4i). The specific capacity was maintained at 551.5 mAh g^−1^ after 2000 cycles with a capacity decay rate of 0.013% per cycle. MnP–Mn_2_P/C@S as a sulfur cathode material exhibits more excellent electrochemical properties than other reported cathodes (Figure S28 and Table S1, Supporting Information). This demonstrates the high specific capacity and excellent cycling stability of MnP–Mn_2_P/C@S cathodes in Li–S batteries.

**Figure 5:**
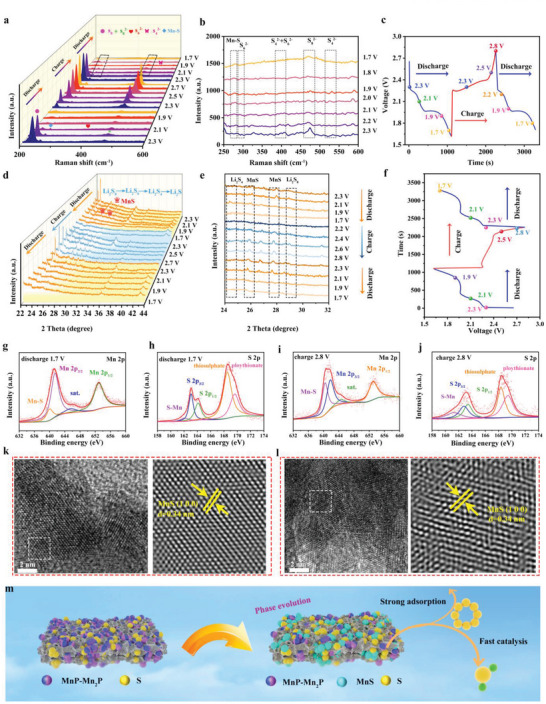
a,b) In situ Raman spectra of MnP–Mn_2_P/C@S during the discharge/charge process at 0.2 C and c) their corresponding discharge/charge curves. d,e) In situ XRD spectra of MnP–Mn_2_P/C@S during the discharge/charge process at 0.2 C and f) the corresponding discharge/charge curves. g) Mn 2p and h) S 2p XPS spectra after discharging to 1.7 V. i) Mn 2p and j) S 2p XPS spectra after charging to 2.8 V. k) HRTEM images of the MnP–Mn_2_P/C@S cathodes for Li–S cells after discharging to 1.7 V and l) HRTEM images of MnP–Mn_2_P/C@S cathodes for Li–S cells after charging to 2.8 V. m) Schematic of the LiPSs conversion behavior on MnP–Mn_2_P/C@S cathodes for Li–S cells.

To further investigate the electrochemical conversion process of MnP–Mn_2_P/C@S cathodes in Li–S batteries, in situ Raman was tested during the discharge/charge process at 0.2 C (**Figure** **5**a). In the initial discharge voltage of 2.3 V, characteristic peaks were observed at 218.65 and 470.4 cm^−1^,^[^ ^38^ ^]^ corresponding to S_8_. As the discharge reaction proceeded, S_8_ was gradually converted to long‐chain LiPSs. During discharge, soluble Li_2_S_n_ (4 ≤ *n* ≤8) characteristic peaks appeared and increased strength. Thereafter, at the voltage of 2.0–1.7 V, Li_2_S*_n_* peaks decreased and disappeared simultaneously due to the gradual conversion of soluble Li_2_S*_n_* to insoluble Li_2_S_2_/Li_2_S, resulting in the appearance of Li_2_S_2_/Li_2_S peaks (360 cm^−1^).^[^ ^39^ ^]^ Notably, an unparalleled characteristic peak appeared at 252 cm^−1^, corresponding to Mn–S^[^ ^40^ ^]^ (Figure 5b,c). These results indicate that the strong affinity between the low‐valence Mn in the MnP–Mn_2_P heterostructure nanoparticles and sulfur atoms in higher order polysulfides formed Mn–S species.^[^ ^41^ ^]^ During charging, insoluble short‐chain Li_2_S_2_/Li_2_S was oxidized into long‐chain LiPSs. When the charging voltage reached 2.8 V, a clear S_8_ peak was noted again at 218.65 cm^−1^, indicating a typical solid–liquid reaction process in the Li–S system. Notably, the characteristic peak of Mn–S was observed throughout the electrochemical reaction. The in situ Raman characteristic peaks showed regular variations, suggesting that MnP–Mn_2_P/C can promote electrochemical behavior and maintain high reversibility.

The in situ XRD exhibits the phase conversion of MnP–Mn_2_P/C@S cathodes in the discharge/charge process, as shown in Figure 5d. During discharge, an S_8_ (PDF#53‐1109) peak^[^ ^42^ ^]^ gradually disappeared and was transformed into LiPSs and later into Li_2_S (PDF#23‐0369).^[^ ^43^ ^]^ Moreover, the characteristic peaks appeared at ≈25.8° and 27.6°, corresponding to MnS (PDF#40‐1288)^[^ ^44^ ^]^ (Figure 5e). This result exhibits the MnS formation on the MnP–Mn_2_P heterostructure nanoparticle surface during the initial discharge process. Li_2_S characteristic peaks (PDF#23‐0369) appeared at ≈27°, which denotes that MnP–Mn_2_P/C accelerated the reaction kinetics. During charging, Li_2_S was converted to Li_2_S_n_ at ≈2.3 V, and the S_8_ peaks gradually appeared at ≈2.8 V (Figure 5f). As the discharge reaction occurred again, the S_8_ peak disappeared, indicating the excellent reversible reaction of MnP–Mn_2_P/C@S cathodes for the Li–S batteries. Notably, despite the significant changes during discharge, the MnS peak remained constant.

To further study the phase conversion of the MnP–Mn_2_P/C@S cathodes, the XPS spectra after discharge and charge were analyzed. When discharging to 1.7 V, the Mn–S characteristic peak appeared at ≈639 eV in the Mn 2p plot (Figure 5g).^[^ ^45^ ^]^ Similarly, the S–Mn characteristic peak appeared at ≈161.5 eV in the S 2p plot (Figure 5h).^[^ ^46^ ^]^ When charging to 2.8 V, Figure 5i shows the presence of the Mn–S characteristic peak (≈639 eV). The S 2p spectra displayed the presence of the S–Mn characteristic peak (≈161.5 eV), as shown in Figure 5j. The high‐resolution S 2p XPS spectra obtained after adsorption also confirmed the presence of the S—Mn bond (Figure S29, Supporting Information). These results indicate the presence of Mn–S species with minimal changes throughout the charging and discharging process.

To verify the presence of MnS and mechanism of the phase conversion in the electrochemical process, the HRTEM spectrum of MnP–Mn_2_P/C@S cathodes after discharge/charge process was obtained (Figure 5k,l). A dot spacing of 0.34 nm, corresponding to the MnS (1 0 0) lattice plane, was obtained by fast Fourier transform. No crystallographic MnS transformation was observed on the surface MnP–Mn_2_P nanoparticles. The generated MnS was widely considered as a booster of polysulfide conversion in Li–S batteries,^[^ ^47^ ^]^ which was beneficial for improving the electrochemical performance of Li–S batteries when combined with MnP–Mn_2_P. In future practical advances in Li–S batteries, the transformation in the phase conversion provides an important direction for improving the reaction kinetics during discharging/charging.

**Figure 6:**
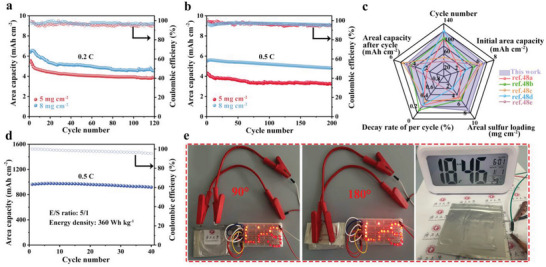
Cycling performances of MnP–Mn_2_P/C@S with the sulfur loadings of 5 mg cm^−2^ and 8 mg cm^−2^ at a) 0.2 C and b) 0.5 C. c) Cycling performances of MnP–Mn_2_P/C@S under high sulfur loading with reported cathodes. d) Cycling performance of MnP–Mn_2_P/C@S║Li flexible Li–S pouch cell at 0.5 C. e) Application of MnP–Mn_2_P/C@S║Li flexible Li–S pouch cell.

To further demonstrate the high sulfur loading of MnP–Mn_2_P/C@S cathodes with MnS phase species, which is crucial in increasing the practical deployment of Li–S batteries, batteries with high sulfur loading (5.0–8.0 mg cm^−2^) were obtained.^[^ ^48^ ^]^ When a sulfur loading of 5 mg cm^−2^ was achieved, the initial area capacity of MnP–Mn_2_P/C@S was 5.5 mAh cm^−2^ at 0.2 C. After 120 cycles, the capacity was 3.9 mAh cm^−2^. MnP–Mn_2_P/C@S exhibited an area capacity of 6.4 mAh cm^−2^ even with a sulfur loading of 8 mg cm^−2^ at a current density of 0.2 C, which was maintained at 4.5 mAh cm^−2^ after 120 cycles (**Figure** **6**a). Moreover, MnP–Mn_2_P/C@S delivered the initial area capacity of 4.3 mAh cm^−2^ with a sulfur loading of 5 mg cm^−2^ and 5.5 mAh cm^−2^ with a sulfur loading of 8 mg cm^−2^ at 0.5 C (Figure 6b). As shown in Figure 6c, this demonstrates the excellent stability of MnP–Mn_2_P/C@S as a sulfur cathode material under high loading than other reported cathodes (Table S2, Supporting Information).^[^ ^49^ ^]^ MnP–Mn_2_P/C@S cathode exhibited outstanding electrochemical properties due to the proper specific surface area in a porous structure and excellent catalytic, adsorptive, and conductive properties. To illustrate the potential of MnP–Mn_2_P/C@S cathodes for actual applications, a Li–S soft pouch cell with MnP–Mn_2_P/C@S was assembled and supplied with an open‐circuit voltage of 2.2 V (Figure S30a, Supporting Information). As shown in Figure 6d, when the area sulfur loading reached 7.3 mg cm^−2^ and the E/S ratio was 5:1, the MnP–Mn_2_P/C@S║Li flexible Li–S pouch cell exhibited great specific capacity (963.8 mAh g^−1^) and excellent energy density (360 Wh kg^−1^) at 0.5 C. Figure 6e; Figure S30b, Supporting Information, show the MnP–Mn_2_P/C@S║Li flexible Li–S pouch cell as the device to light a single diode lamp consisting of a “Li–S” at various bends (0°, 90°, 180°).^[^ ^50^ ^]^ An electric fan with a clock was powered by a single coin cell as a practical illustration of MnP–Mn_2_P/C@S║Li flexible Li–S pouch cells. This shows that Li–S batteries with MnP–Mn_2_P/C@S cathode have strong potential for actual production.

## 3 Conclusion

In this study, we designed MnP/C, Mn_2_P/C, and MnP–Mn_2_P/C cathodes with various stoichiometries via a simple route by controlling the manganese species concentration as sulfur hosts for Li–S batteries. The experimental results and DFT calculation data exhibit the suitability of MnP–Mn_2_P/C for the effective adsorption of LiPSs and excellent electrocatalytic activity, compared with MnP/C and Mn_2_P/C. Based on these characteristics, ultra‐high initial specific capacities of 1384.7 and 763.3 mAh g^−1^ were obtained at current densities of 0.2 and 5.0 C, respectively. With a low capacity fading of 0.013% per cycle over 2000 cycles and excellent rate capability of up to 5.0 C, MnP–Mn_2_P/C@S cathodes provided outstanding long‐term cycling stability. In addition, we discovered the novel phase conversion mechanism of MnP–Mn_2_P/C heterostructure particles in a polysulfide conversion process and production of MnS by the electrochemical transition. Based on the combination of surface reaction layers and polysulfides, we realized stable cycling and superior capacity sulfur cathodes at high loadings. Therefore, compared with MnP/C, Mn_2_P/C, and MnP–Mn_2_P/C heterostructure cathodes with optimized phase for Li–S batteries are a promising material with strong potential for actual applications.

## Conflict of Interest

The authors declare no conflict of interest.

## Supporting information

Supporting Information

## Data Availability

The data that support the findings of this study are available from the corresponding author upon reasonable request.
