# Supplementary material for: Li–S Chemistry of Manganese Phosphides Nanoparticles With Optimized Phase
Source: Adv Sci (Weinh). 2023 Feb 3;10(9):2207470. doi: 10.1002/advs.202207470 (PMC10037994; doi:10.1002/advs.202207470)
Supplement: Supplementary file 1 — Supporting Information [file ADVS-10-2207470-s001.pdf]

ADVANCED SCIENCE
Open Access

Supporting Information

for *Adv. Sci.*, DOI 10.1002/advs.202207470

Li–S Chemistry of Manganese Phosphides Nanoparticles With Optimized Phase

*Qiao Deng, Xinji Dong, Pei Kang Shen and Jinliang Zhu**

Supporting Information

Li–S chemistry of manganese phosphides nanoparticles with optimized phase

*Qiao Deng, Xinji Dong, Pei Kang Shen, Jinliang Zhu**

Experimental Section

Synthesis of MnP/C, Mn_2P/C and MnP–Mn_2P/C

250 mL of 0.05 mmoL manganese acetate tetrahydrate (Shanghai Aladdin Biochemical Technology Co., Ltd., China) was added to a beaker containing 50 g of crushed phosphorus–containing resin (Tianjin Bohong Resin Technology Co., Ltd., China), stirred at 60 °C for 360 min, filtered and the clean filtered resin were dried at 75 °C for 24 h. Afterward, the dried resin was combined with 10 g of KOH (analytical Ltd.) into a ball mill tank (Planetary Ball Mill, Changsha MiQi Technology Co., Ltd.) for 30 min. The ball-milled mixture was placed in a tube furnace and pyrolyzed at ～1100 °C for 60 min under a nitrogen atmosphere and cooled to room temperature at a rate of 10 °C min^{-1}. Then, the pyrolysis product was placed in deionized water, washed several times with deionized water, sonicated for 20 min, and filtered several times with deionized water until the pH of the filtrate was closing to neutral. The collected solid filtrate was dried at 75 °C for 12 h. The dried solid powder was MnP–Mn_2P/C. 5 g and 15 g of KOH were added instead of 10 g of KOH as described above to obtain MnP/C and Mn_2P/C, respectively. Inductively coupled plasma–atomic emission spectrometry (ICP) and EDS analysis exhibited that the MnP, MnP–Mn_2P, and Mn_2P contents within the prepared samples were 42.4, 48.4, and 54.9 wt%, respectively. MnP content in the MnP-Mn_2P/C composite is 19.6 wt%.

Synthesis of MnP–Mn_2P/C@S

MnP–Mn_2P/C and sulfur powder (99.98 wt%, Sigma-Aldrich) were mixed with a mass ratio of 1:4 and the mixtures were ball milled for 30 minutes. Then the mixtures were transferred to a hydrothermal reactor lined with Teflon, where it was heated to 155 °C and reacted for 12 h.

Physical characterization

The X-ray diffractometer was a D/Max-III X-ray diffractometer (Rigaku Co., Japan) with CuK-α irradiation at 40 kV and 30 mA. The Raman spectroscopy was performed on a Raman spectrometer with a 532 nm He/Ne laser (Horiba Jobin Yvon Inc, France). The specific surface area and pore size distribution were analyzed using an ASAP 2460 specific surface area analyzer (Micromeritics Co., USA). The S content was measured with a thermogravimetric analyzer (DSC/TGA; Netzsch STA449 F5 Jupiter) at 30-850 °C in nitrogen. Microstructures were characterized by a field emission scanning electron microscope (SU8220, Hitachi, Japan) and a transmission electron microscope (Tian Etem G2 80-300, FEI, USA). X-ray photoelectron spectroscopy was carried out using an ESCALAB 250 energy spectrometer with a monochromatic Al Kα radiation source. The electrical conductivity of the samples was tested using an ST-2722 semiconductor resistivity tester (Suzhou Jingge Electronic Co., Ltd.). UV-vis absorption spectrum was tested using a UV-vis spectrophotometer (PerkinElmer Lambda 650, USA).

Prepation of MnP–Mn_2P/C@S cathode

The electrode slurry was prepared by dispersing 80 wt% of active material (MnP–Mn_2P/C@S nanocomposite), 10 wt% of Super-p conductive agent, and 10% of polyvinylidene fluoride (PVDF) binder in *N*-methyl pyrrolidone. The slurry was poured onto aluminum foil with a squeegee and dried in an oven at 55 °C for 12 h. The aluminum foil coated with the active material was stamped with a press to form a 14 mm electrode sheet with loading of approximately 1.8 mg cm^{-2}.

Visual observation of polysulfide adsorption

A mixture of sulfur and lithium sulfide in a 1:3 ratio of substance was added to a solvent mixture of 1,2-dimethoxyethane/1,3-dioxopentane (DME/DOL, 1:1 (v/v)) and stirred at 60 °C for 24 h to give a 3 M solution of Li_2S_6. 20 mg of active material were added to 4 mL of Li_2S_6 solution and photographs were taken at room temperature after 5 h. Afterward, the adsorption properties of the three active materials were tested by UV-vis spectrophotometry.

Assembly of symmetric cells

To prepare the electrodes for the symmetrical cells, MnP–Mn_2P/C@S, Mn_2P/C@S, and MnP/C@S were homogeneously mixed with polyvinylidene fluoride (PVDF) in a 9:1 mass ratio in *N*-methyl pyrrolidone to form the electrode slurry, which was evenly coated onto aluminum foil with a squeegee. Two identical electrode sheets were assembled into 2032 button

cells with 0.3 M, 30 μL of Li_2S_6. Cyclic voltammetric curves were measured with an IM6 electrochemical workstation (Zahner-Elektrik, Germany) at a scan rate of 5 mV s^{-1} and voltages in the range of -0.8 V to 0.8 V.

Nucleation of Li_2S

Li_2S and S in a molar ratio of 1:7 were dissolved in a (DOL/DME (1:1 (v/v)) solvent mixture containing 1.0 M lithium (bis(trifluoromethanesulfonyl)imide) (LiTFSI) and stirred at 80 °C to produce a Li_2S_8 solution. The MnP–Mn_2P/C@S sample was suspended in ethanol and the suspension was added dropwise to a 14 mm diameter carbon cloth dried at 60 °C for 12 h, then a working electrode was obtained. A lithium foil was used as the counter electrode, a Celgard 2400 septum as the separator, and a carbon cloth loaded with MnP–Mn_2P/C@S as the working electrode, which a Li_2S_8 cathode solution (30 μL) was added dropwise. A blank electrolyte of 30 μL of 1 wt% 30 μL $LiNO_3$ and no Li_2S_8 was added to the working electrode. The cell was placed on a battery test system (Shenzhen Newwere Battery Co) and discharged at a constant current of 0.112 mA and a voltage of 2.11 V, after which the cell voltage was kept at 2.1 V to measure the nucleation process.

Electrocatalytic activity evaluation

The electrocatalytic activity evaluation was obtained using a three-electrode system with controlled valence at an electrochemical work station (Pine Instrument Co., USA). The counter electrode and the reference electrode were each lithium foil. The working electrode was a rotating disc electrode. The rotating disc electrode, also known as a glassy carbon electrode, has an area of 0.196 cm^{-2}. (Tianjin ida Heng Sheng Technology Co.)

The electrocatalyst was prepared by drop-in injection onto a disc electrode. 5.0 mg of electrocatalyst was dispersed in a mixture of 1 mL of isopropanol, 50 μL of Nafion (0.5 wt%, DuPont, USA), and the resulting mixture was sonicated for 30 minutes to form a homogeneous slurry. The rotating disc electrodes were ultrasonically cleaned with ethanol and deionized water, mechanically washed, and 4 μL of the suspension was dropped onto the rotating disc electrodes. After evaporation of the solvent, a working electrode was successfully prepared. The surface loading of the electrocatalyst on the working electrode was approximately 0.1 mg cm^{-2}.

The tests were performed on 4 mmoL of S_8 and 1.0 M LiTFSI dissolved in DOL/DME (1:1 (v/v)). Li^+/Li was scanned throughout the tests using linear scanning voltammetry in the range

of 3-1.60 V at a rate of 10 mV s^{-1} at 1000 rpm. The Tafel slope was obtained from the LSV curve and the Tafel equation

$$V=a+b\log j \quad (1)$$

V represents voltage, b represents Tafel slope and *j* represents rotating disc current density.

Li–S Pouch Cell Assembly

MnP–Mn_2P/C@S was used as the sulfur cathode and the lithium strip as the anode, each cut into rectangular pieces of 5.5×4.0 cm. An area sulfur content of Li–S pouch cell was approximately 7.3 mg cm^{-2}. The E/S ratio was approximately 5:1 and the thickness of the lithium strip anode was approximately 0.4 mm.

Measurement of the galvanostatic intermittent titration (GITT)

MnP–Mn_2P/C@S was used as the sulfur cathode and the lithium strip as the anode. A current pulse at 0.05 C for 10 minutes is followed by 20 minutes of rest between 1.7 and 2.8 V (vs Li^+/Li). QOCV represents open-circuit voltage, CCV represents closed-circuit voltage.

Density functional theory calculation

First-principles calculations were performed within Vienna Ab initio Simulation Package (VASP). The GGA calculation was carried out within the Perdew-Burke-Ernzerhof (PBE) exchange-correlation potential. The DFT+D3 with Becke-Johnson damping was applied to describe the vander Waals interaction between layers. the cut-off energy is used to be 500 eV; the positions of all atoms and the cell parameters were relaxed until the average force per atom decreases to 0.01 eV/Å; the Brillouin zone integration was carried out by selecting a k-point mesh satisfied ka~30 A in different models. All calculations were spin-polarized. The binding energy of S_8 and Li_2S_x adsorbed on different surfaces were calculated in MnP (0 2 0) surface and Mn_2P (2 0 0)surface. The binding energy was calculated by the equation

$$\Delta E=E_{(slad+ads)}-E_{(slad)}-E_{(ads)} \quad (2)$$

$$\Delta G_{(ads)}=\Delta E_{(ads)}-\Delta ZPE-T\Delta S \quad (3)$$

Whereas ΔE_{ads} was the difference in DFT energy before and after adsorption, ΔG_{ads} was the free energy difference before and after adsorption, ΔZPE was the Zero point energy difference before and after adsorption and ΔS was the difference in entropy before and after adsorption.

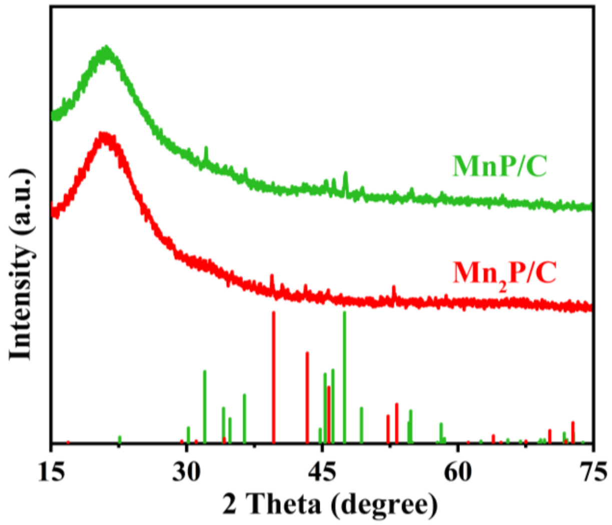

Figure S1. The XRD patterns of MnP/C and Mn_2P/C.

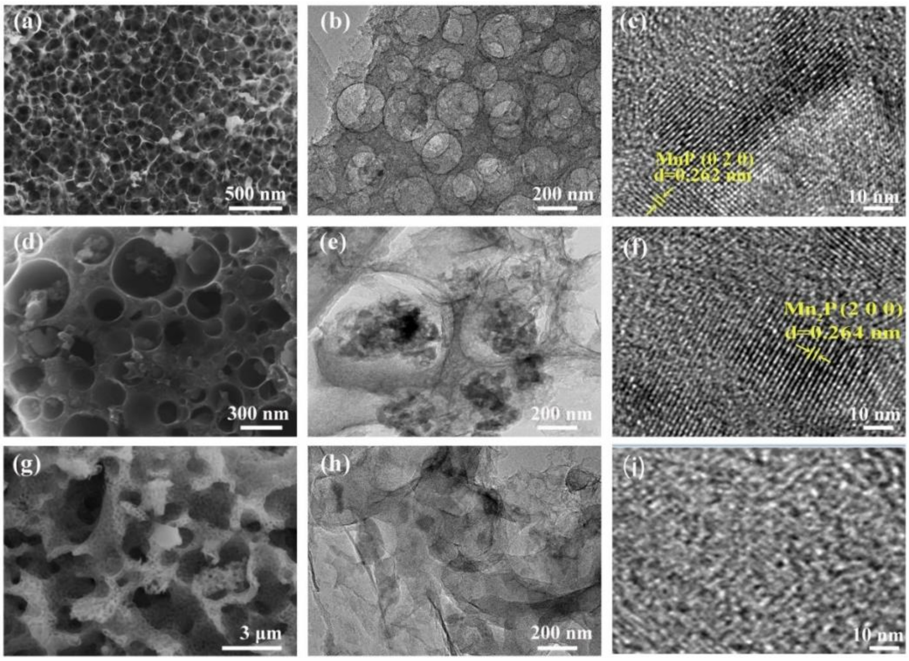

Figure S2. SEM, TEM, and HRTEM images of a-c) MnP/C, d-f) Mn_2P/C, and g-i) synthesized carbon.

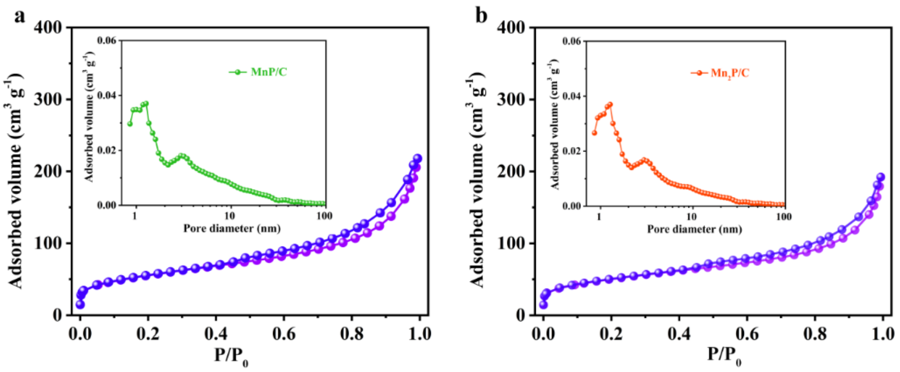

Figure S3. N_2 adsorption/desorption analysis of a) MnP/C and b) Mn_2P/C.

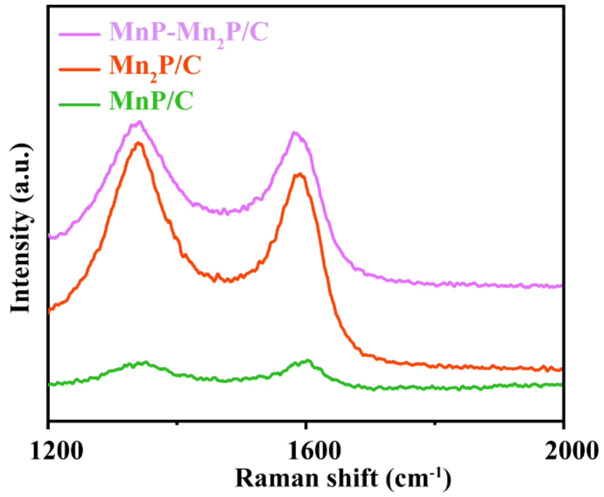

Figure S4. Raman patterns of MnP–Mn_2P/C, Mn_2P/C, MnP /C.

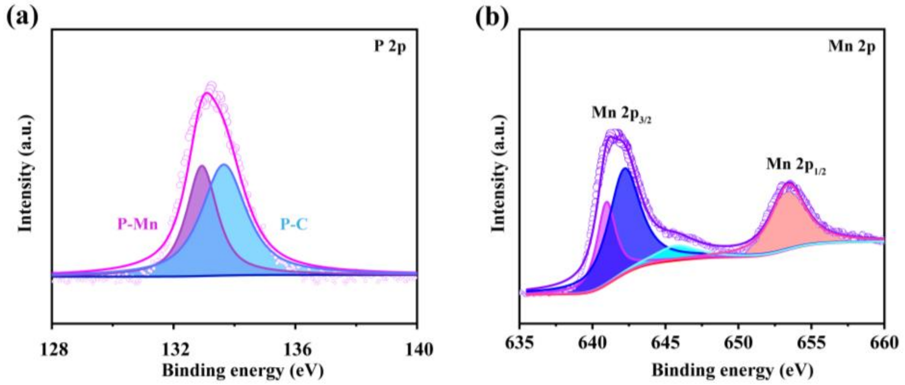

Figure S5. a) XPS-P 2p and b) Mn 2p spectra of MnP–Mn_2P/C.

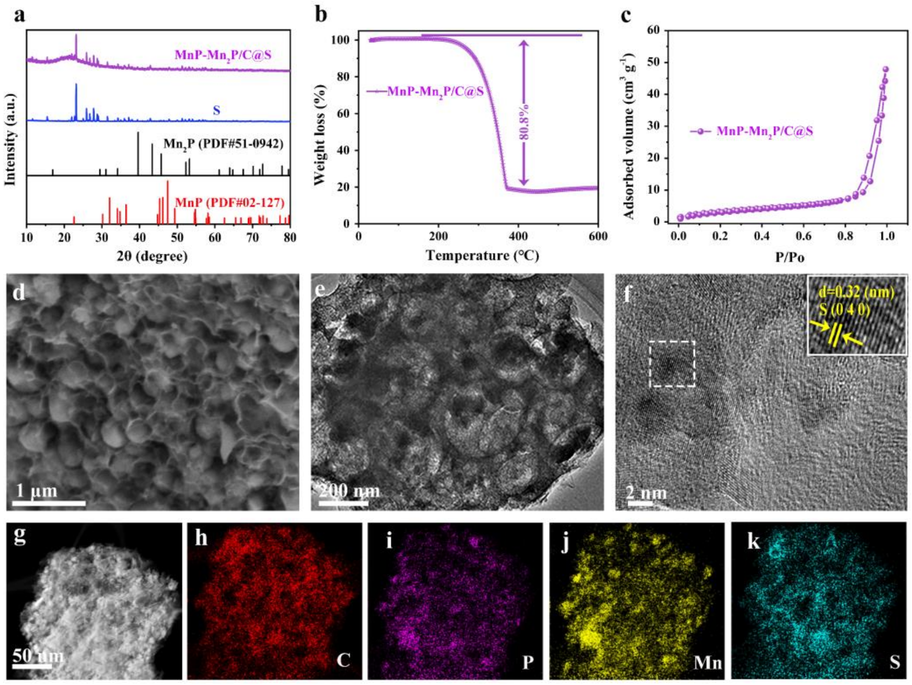

Figure S6. a) XRD spectra, b) TGA curves, c) N_2 adsorption/desorption analysis, d) SEM image, e) TEM image, f) HRTEM image, g) STEM, and h–k) EDX element distribution mappings of MnP–Mn_2P/C@S.

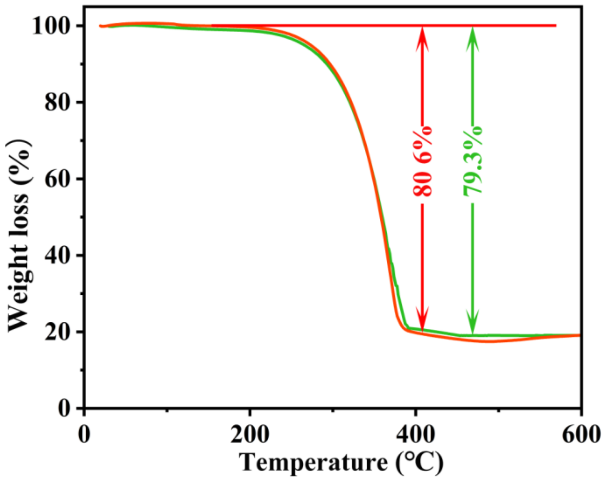

Figure S7. TG curves of Mn_2P/C@S, and MnP/C@S.

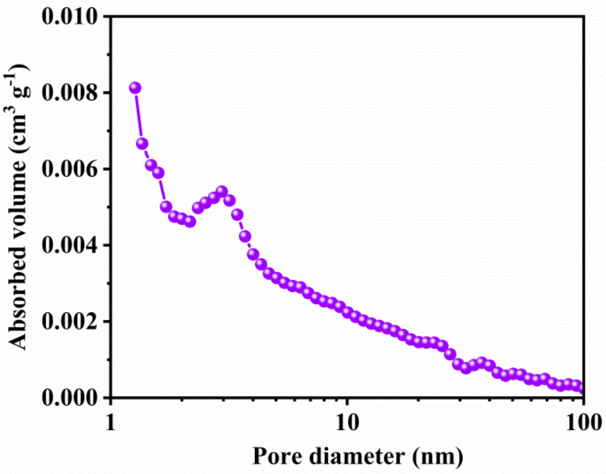

Figure S8. The pore size distribution of MnP–Mn_2P/C@S.

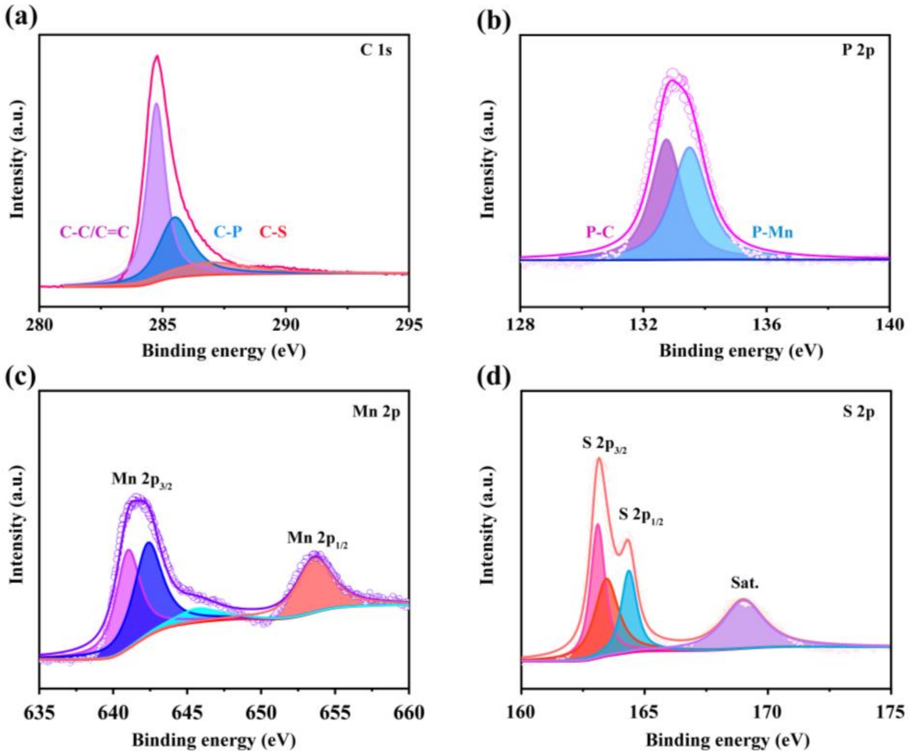

Figure S9. XPS- a) C 1s, b) P 2p, c) Mn 2p and d) S 2p of MnP–Mn_2P/C@S.

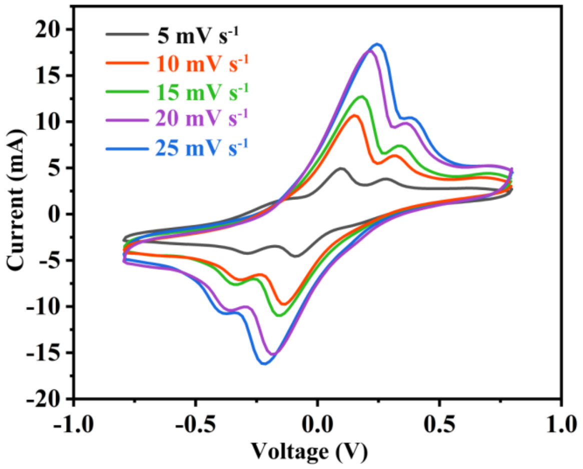

Figure S10. Typical CV curves of symmetric cells based on MnP–Mn_2P/C.

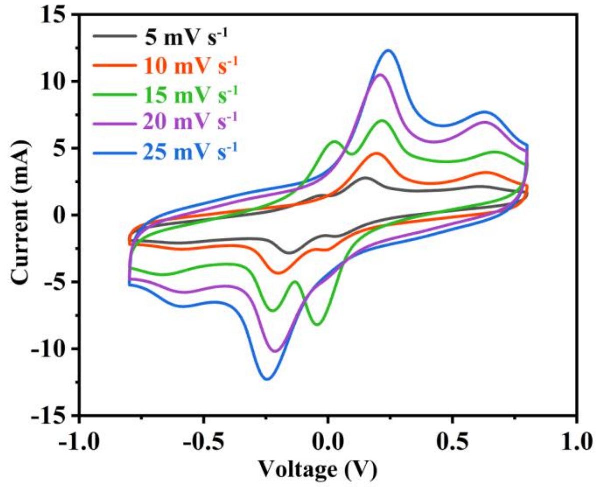

Figure S11. Typical CV curves of symmetric cells based on MnP/C.

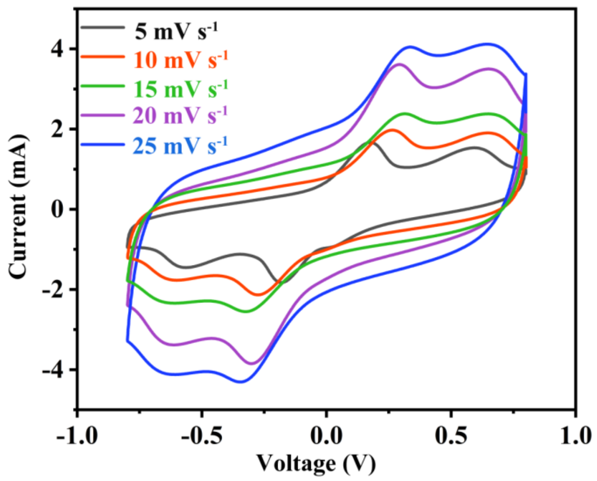

Figure S12. Typical CV curves of symmetric cells based on Mn_2P/C.

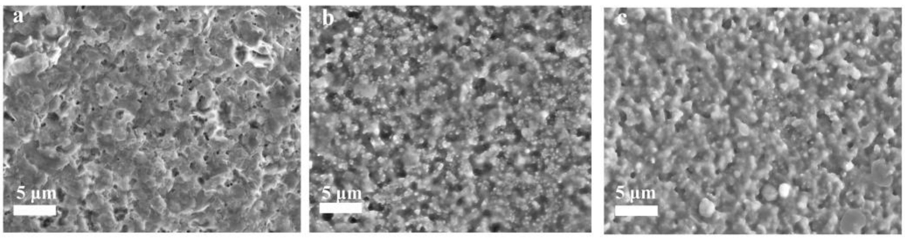

Figure S13. SEM images after Li_2S nucleation of a) MnP–Mn_2P/C, b) MnP/C, c) Mn_2P/C.

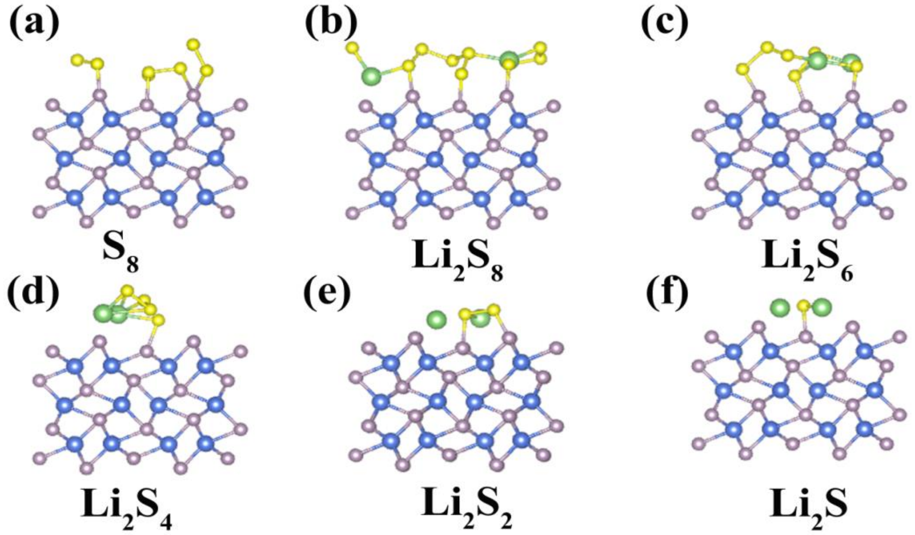

Figure S14. The DFT theorital calculation of MnP/C.

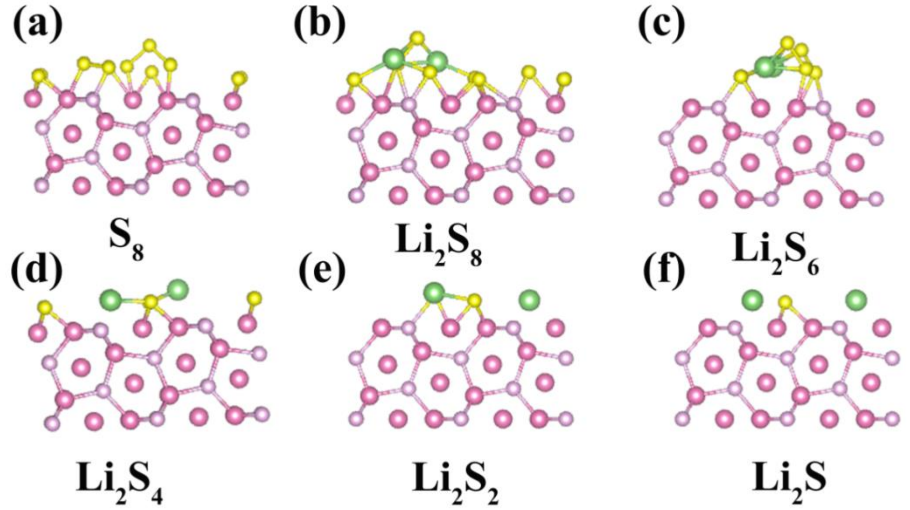

Figure S15. The DFT theorital calculation of Mn_2P/C.

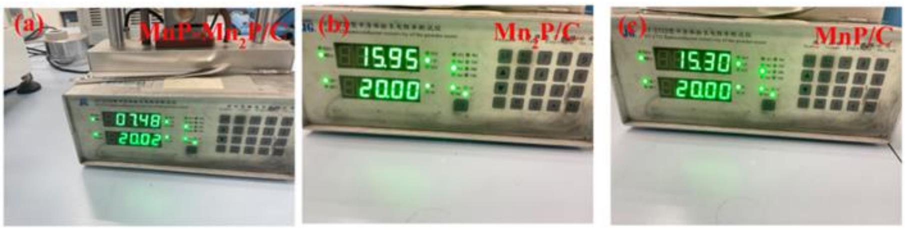

Figure S16. a-c) Conductivity of MnP–Mn_2P/C, Mn_2P/C, and MnP/C.

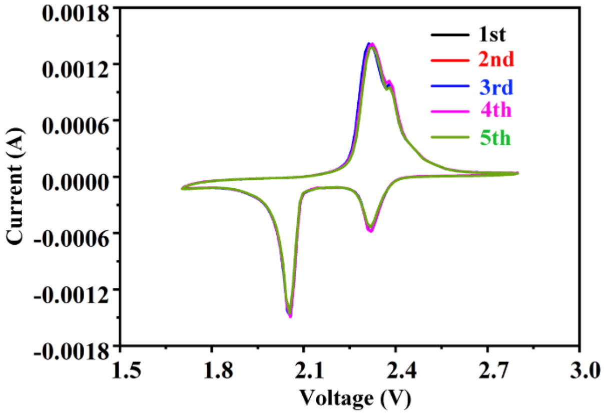

Figure S17. The CV curves of MnP–Mn_2P/C@S with 0.1 mV s^{-1}.

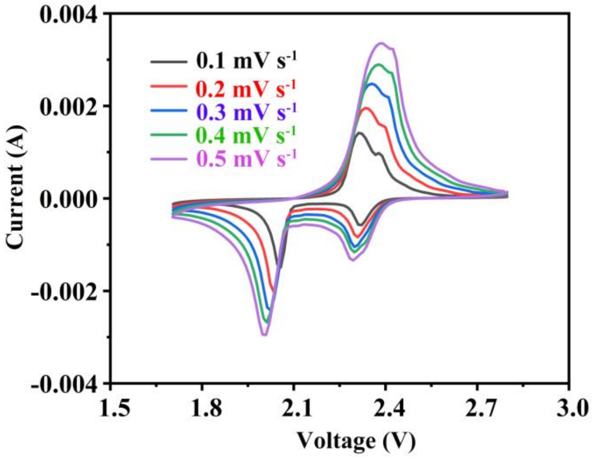

Figure S18. The CV curves of MnP–Mn_2P/C@S.

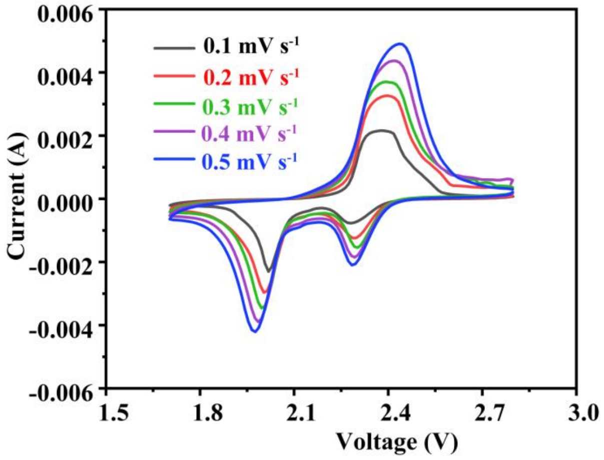

Figure S19. The CV curves of MnP/C@S.

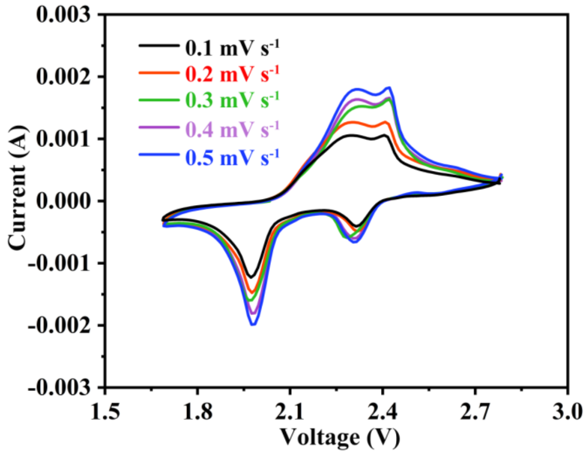

Figure S20. The CV curves of Mn_2P/C@S.

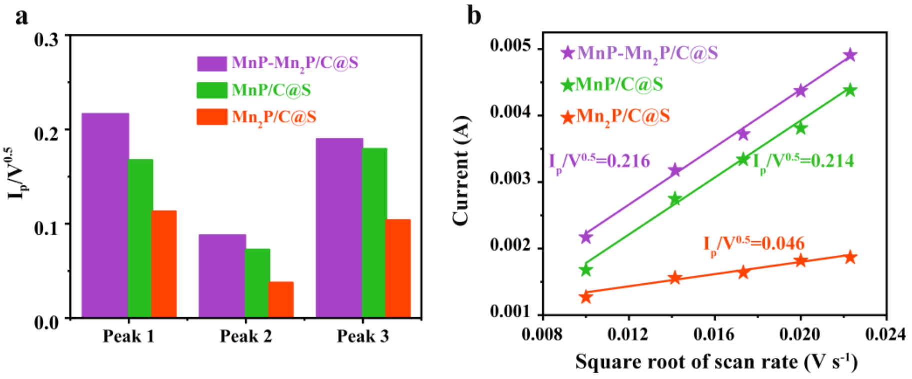

Figure S21. a-b) lithium ion diffusion rate for MnP–Mn_2P/C@S, Mn_2P/C@S, and MnP/C@S.

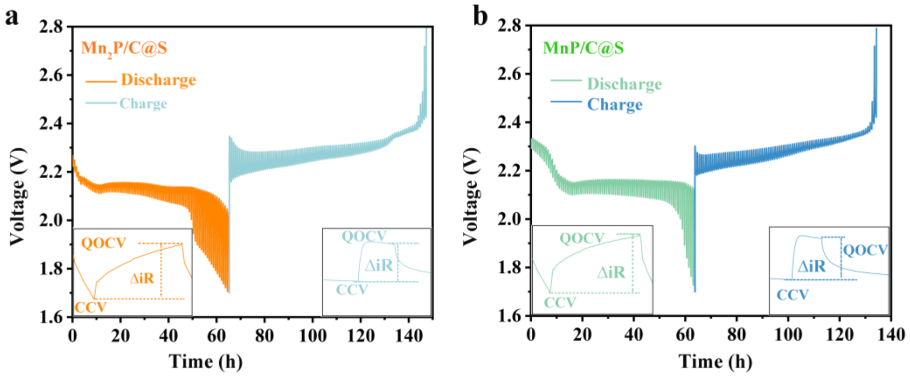

Figure S22. GITT of a) Mn_2P/C@S and b) MnP/C@S.

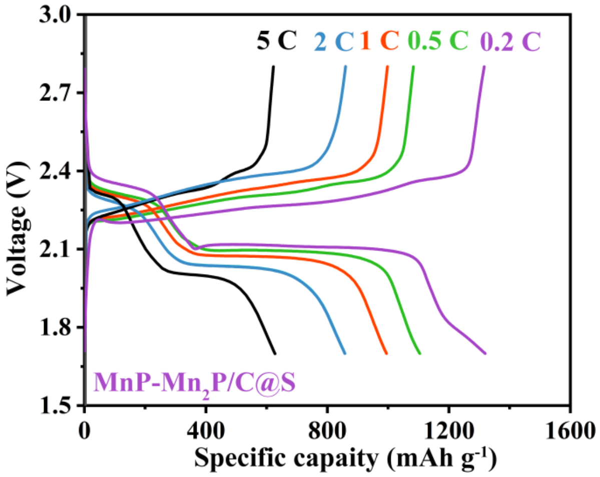

Figure S23. Charge–discharge profiles of MnP–Mn_2P/C@S cells at various rates.

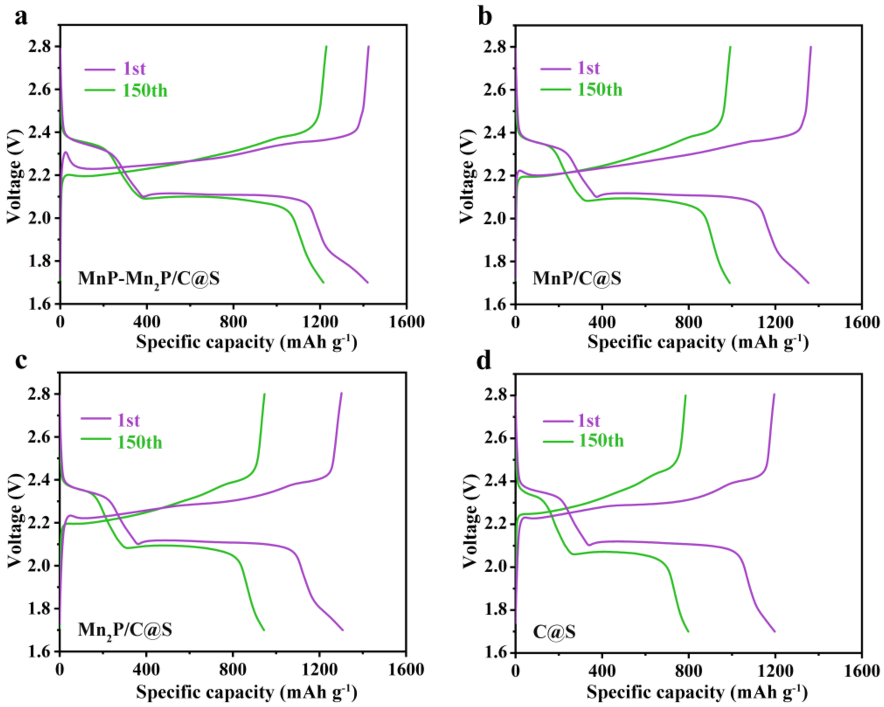

Figure S24. Charge–discharge profiles before and after cycling of a) MnP–Mn_2P/C@S, b) MnP/C@S, c) Mn_2P/C@S, and d) C@S.

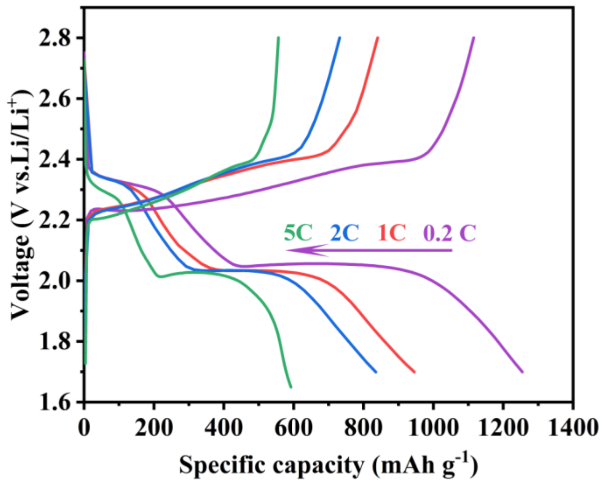

Figure S25. Charge–discharge profiles of MnP/C@S.

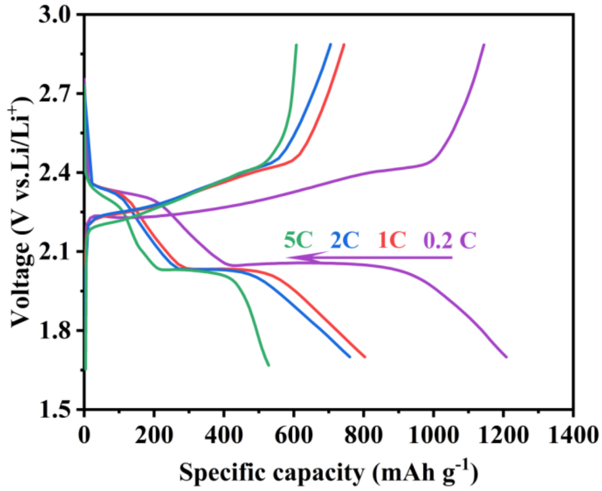

Figure S26. Charge–discharge profiles of Mn_2P/C@S.

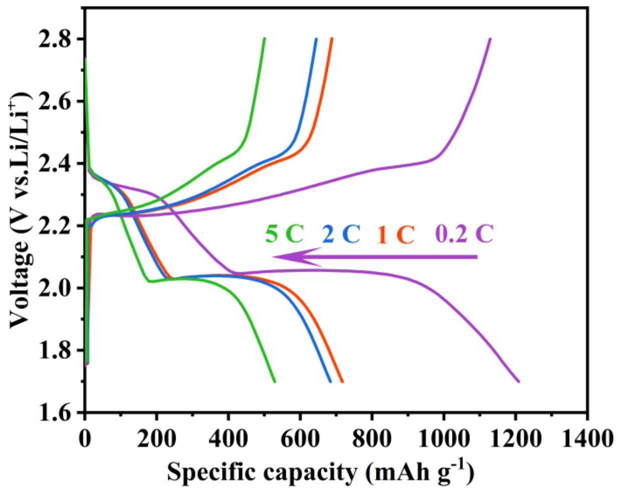

Figure S27. Charge–discharge profiles of C@S

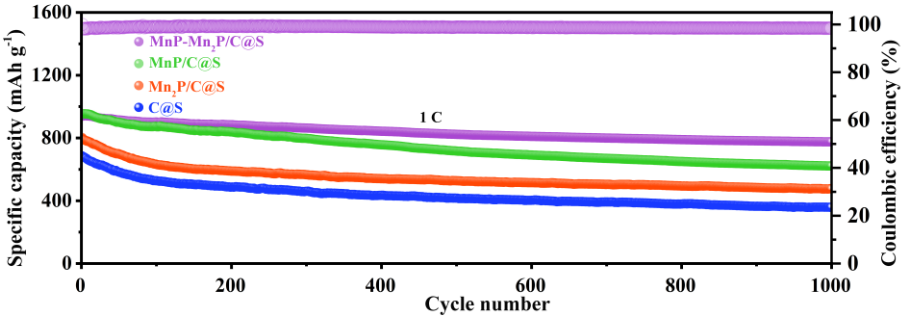

Figure S28. Long–term cycling performance of MnP–Mn_2P/C@S, Mn_2P/C@S , MnP/C@S, and C@S for 1000 cycles at 1 C.

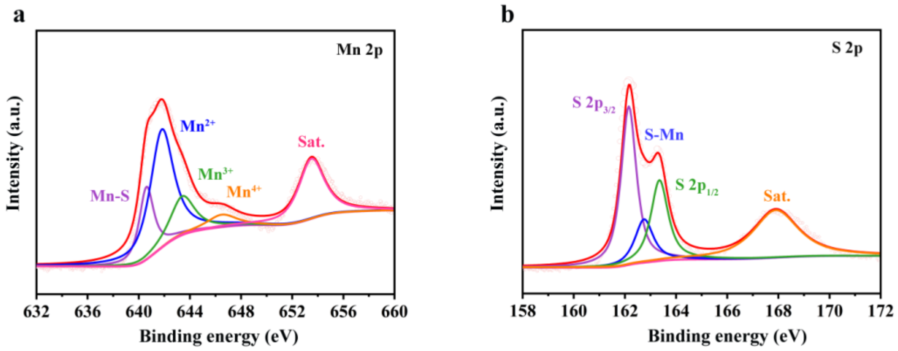

Figure S29. XPS- a) Mn 2p, b) S 2p for MnP–Mn_2P/C after adsorption.

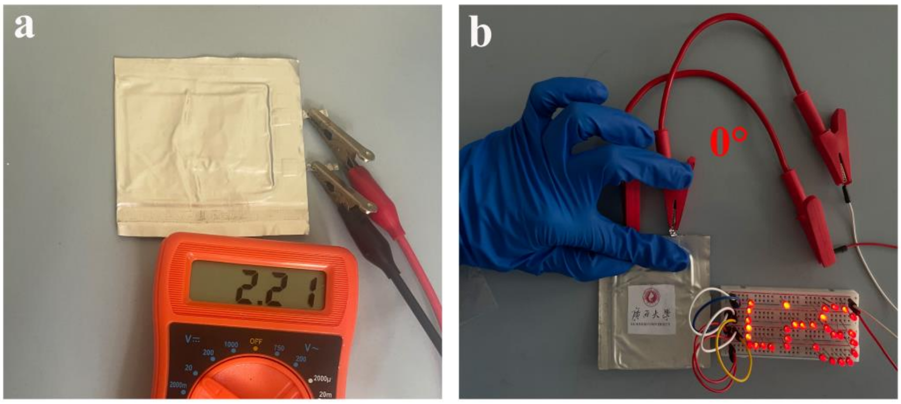

Figure S30. a) The open–circuit voltage of MnP–Mn_2P/C@S║Li flexible Li–S pouch cell, b) application of flexible Li–S pouch cell.

Table S1. Comprehensive comparison of electrochemical performances among MnP–Mn_2P/C and reported hosts for Li–S batteries.

Host materials	Sulfur content (wt%)	Sulfur loading (mg cm^{-2})	Rate (C)	Initial capacity (mAh g^{-1})	Cycle number	Capacity after cycle (mAh g^{-1})	Decay rate of per cycle (%)
**MnP–Mn_2P/C (This work)**	80%	1.8	5	763.3	2000	551.5	0.013
Mn/C(N,O)[1]	76%	1.3	1	900	1000	450	0.05
ON–MnPC[2]	49%	1.7~2.0	0.2	712.3	100	637.5	0.11
rGO–$SmMn_2O_5$[3]	70%	1	0.5	891	1500	333.2	0.316
MnO/PPy–3[4]	60%	1.5	1	848	1000	580	0.032
Mn/Co–N-C[5]	75%	0.6	2	816	1000	622	0.036
MnS/CNF[6]	77.9%	2.0	1	810	400	648	0.05
NS-core/MnO_2[7]	85%	1.5	2	900	1700	315	0.038
Ni_2P–HCS[8]	70%	1.5	1	926.4	500	781.8	0.037
FeP/rGO/CNTs[9]	75%	1	1	780.6	400	672.9	0.04
rGO–CNT–CoP(A)[10]	67%	2.1	2	728	200	617	0.09

Table S2. Comprehensive comparison of electrochemical performances among MnP–Mn_2P/C based on high sulfur loadings and reported hosts in Li-S batteries.

Host materials	Sulfur loading (mg cm^{-2})	Rate (C)	Initial capacity (mAh cm^{-2})	Cycle number	Capacity after cycle (mAh g^{-1})	Decay rate of per cycle (%)
**MnP–Mn_2P/C (This work)**	5.0<br>8.0	0.5<br>0.2	4.3<br>6.4	200<br>120	3.7<br>4.5	0.07<br>0.23
h–Mn–N–C[11]	4.9	0.2	2.68	100	2	0.26
CoP[12]	3.0	0.5	4.3	100	3.7	0.14
Co–Fe–P[13]	5.5	0.2	4.8	100	4	0.17
rGO/CoP[14]	5.2	0.2	5.9	50	4.9	0.33
MnS/CNF[6]	5	0.5	6	200	4.5	0.13
MoP–CNT[15]	6	0.8	5	50	4.5	0.2
MoP_2@BP[16]	2.8	0.2	3.74	120	2.52	0.27
CNT–CoP–Vp[17]	4.7	0.2	5.65	40	4.18	0.65
FeP/rGO/CNTs[9]	3.5	1	4.2	200	2.4	0.21

References

[1] L. Wang, W. Hua, X. Wan, Z. Feng, Z. Hu, H. Li, J. Niu, L. Wang, A. Wang, J. Liu, X. Lang, G. Wang, W. Li, Q. H. Yang, W. Wang, *Adv. Mater.* **2022**, 34, 2110279.

[2] H. Yu, P. Zeng, X. Zhou, C. Guo, X. Liu, K. Wang, X. Guo, B. Chang, M. Chen, X. Wang, *ACS Appl. Mater. Interfaces* **2021**, 13, 54113.

[3] Y. Liu, Z. Wei, B. Zhong, H. Wang, L. Xia, T. Zhang, X. Duan, D. Jia, Y. Zhou, X. Huang, *Energy Stor. Mater.* **2021**, 35, 12.

[4] Y. Feng, H. Liu, Q. Lu, Y. Liu, J. Li, X. He, X. Liu, D. Mikhailova, *J. Power Sources* **2022**, 520, 230885.

[5] S. Qiao, Q. Wang, D. Lei, X. Shi, Q. Zhang, C. Huang, A. Liu, G. He, F. Zhang, *J. Mater. Chem. A* **2022**, 10, 11702.

[6] X. Wang, X. Zhao, C. Ma, Z. Yang, G. Chen, L. Wang, H. Yue, D. Zhang, Z. Sun, *J. Mater. Chem. A* **2020**, 8, 1212.

[7] X. Liang, L. F. Nazar, *ACS Nano* **2016**, 10, 4192.

[8] S. Yang, R. Xiao, T. Hu, X. Fan, R. Xu, Z. Sun, B. Zhong, X. Guo, F. Li, *Nano Energy* **2021**, 90, 106584.

[9] S. Huang, Y. V. Lim, X. Zhang, Y. Wang, Y. Zheng, D. Kong, M. Ding, S. A. Yang, H. Y. Yang, *Nano Energy* **2018**, 51, 340.

[10] R. Sun, Y. Bai, M. Luo, M. Qu, Z. Wang, W. Sun, K. Sun, *ACS Nano* **2021**, 15, 739.

[11] S. Qiao, D. Lei, Q. Wang, X. Shi, Q. Zhang, C. Huang, A. Liu, G. He, F. Zhang, *Chem. Eng. J.* **2022**, 442, 136258.

[12] C. Qi, Z. Li, C. Sun, C. Chen, J. Jin, Z. Wen, *ACS Appl. Mater. Interfaces* **2020**, 12, 49626.

[13] Y. Chen, W. Zhang, D. Zhou, H. Tian, D. Su, C. Wang, D. Stockdale, F. Kang, B. Li, G. Wang, *ACS Nano* **2019**, 13, 4731.

[14] J. Zhou, X. Liu, L. Zhu, J. Zhou, Y. Guan, L. Chen, S. Niu, J. Cai, D. Sun, Y. Zhu, J. Du, G. Wang, Y. Qian, *Joule* **2018**, 2, 2681.

[15] Y. Yang, Y. Zhong, Q. Shi, Z. Wang, K. Sun, H. Wang, *Angew. Chem. Int. Ed.* **2018**, 57, 15549.

[16] Y. Luo, N. Luo, W. Kong, H. Wu, K. Wang, S. Fan, W. Duan, J. Wang, *Small* **2018**, 14, 1702853.

[17] R. Sun, Y. Bai, Z. Bai, L. Peng, M. Luo, M. Qu, Y. Gao, Z. Wang, W. Sun, K. Sun, *Adv. Energy Mater.* **2022**, 12, 2102739.
